# Problematic Smartphone Use and Adolescent Developmental Outcomes: A Three-Wave Longitudinal Analysis of Prospective Associations Through Academic Stress and Time-Use Pathways

**DOI:** 10.3390/bs16071224

**Published:** 2026-07-18

**Authors:** Joungmin Kim

**Affiliations:** College of Hyangseol Liberal Arts, Healthcare Service School, Soonchunhyang University, 22 Soonchunhyang-ro, Asan-si 31538, Republic of Korea; nicki123@sch.ac.kr; Tel.: +82-42-530-4874

**Keywords:** problematic smartphone use, adolescence, longitudinal mediation, academic stress, self-regulated learning, ego resilience, subjective happiness, Panel Study on Korean Children, Monte Carlo confidence interval

## Abstract

Problematic smartphone use in adolescence has been linked cross-sectionally to poorer academic and emotional functioning, but the longitudinal mechanisms remain unclear. Using three waves of the Panel Study on Korean Children (W14–W16; ages 14–16), this study tested whether problematic smartphone use at age 14 predicts four developmental outcomes at age 16—school adjustment, self-regulated learning, ego resilience, and subjective happiness—through four age-15 mediators: gaming, video-watching, self-study hours, and academic stress. Hierarchical regressions with autoregressive controls, bootstrap a-paths, and 16 indirect effects tested with 20,000-draw Monte Carlo confidence intervals were estimated. Problematic smartphone use predicted three outcomes but not subjective happiness, for which the association was indirect only. Seven indirect effects were significant, and six survived Benjamini–Hochberg false discovery rate correction. The findings indicated two pathways: a time-displacement pathway (gaming and reduced self-study) specific to self-regulated learning and an academic-stress pathway linked to school adjustment, ego resilience, and happiness. Crucially, in baseline-controlled sensitivity analyses, the time-use pathways remained significant, whereas the stress pathway was fully attenuated, indicating a concurrent rather than a prospective association. Results were robust across path-model SEM, full-information maximum likelihood, and inverse-probability weighting, supporting a dual-pathway account of adolescent digital development.

## 1. Introduction

### 1.1. Problematic Smartphone Use and Adolescent Development

National statistics from the Ministry of Science and ICT (2023) indicate that more than 40% of Korean adolescents aged 10–19 are at risk of smartphone overdependence, a proportion that has continued to increase over the past decade. As smartphones have become central platforms for entertainment, learning, social interaction, and identity development, growing scholarly attention has focused on whether problematic smartphone use exerts lasting consequences for adolescent development beyond momentary screen exposure (Twenge & Campbell, 2018; Kwak et al., 2018; Sohn et al., 2019; Busch & McCarthy, 2021; Augner et al., 2022). Recent meta-analyses have reported small-to-moderate associations between problematic smartphone use and a range of adverse outcomes, including depression, anxiety, sleep impairment, and poorer academic performance across diverse cultural contexts (Augner et al., 2022; Sohn et al., 2019). However, the causal direction of these associations and their developmental timing remain subjects of ongoing debate (Orben, 2020; Odgers & Jensen, 2020).

Despite a rapidly expanding body of research, a central question remains unresolved: whether problematic smartphone use constitutes a general developmental risk factor or operates through distinct, domain-specific pathways. Recent systematic reviews and meta-analyses have reported associations between problematic smartphone use and mental health or sleep-related outcomes among children and adolescents (Sohn et al., 2019; Yang et al., 2021). This inconsistency raises the possibility that problematic smartphone use does not uniformly influence all aspects of adolescent development but rather operates through differentiated mechanisms that vary by outcome domain.

Cross-sectional studies have repeatedly linked problematic smartphone use with diminished psychological, academic, and behavioral functioning in adolescents, including elevated stress and depression, lower academic achievement, poorer sleep, and impaired self-regulation (Lin et al., 2015; H. J. Kim et al., 2017; Rosen et al., 2013; Yang et al., 2021). However, cross-sectional designs cannot disentangle temporal ordering or identify the processes through which these associations unfold. As a result, much of the existing literature documents correlations without specifying how, when, or for whom problematic smartphone use becomes developmentally consequential. Despite this consistent evidence, longitudinal studies that can specify mechanisms remain comparatively scarce.

### 1.2. A Dual-Mechanism Theoretical Framework

This study posits that problematic smartphone use in early adolescence influences later developmental outcomes through two complementary mechanisms, grounded in developmental theory.

The first is a stress-activation mechanism, consistent with Lazarus and Folkman’s (1984) transactional model of stress. To the extent that problematic smartphone use interferes with sleep, study routines, and offline social engagement, it generates secondary appraisals of academic demands that exceed personal coping resources (Yang et al., 2021). Elevated academic stress, in turn, is well documented to undermine school adjustment, emotional resilience, and subjective happiness in adolescents (Jiang, 2021; Lee & Lee, 2021; Steare et al., 2023). In Korean adolescents specifically, the convergence of high academic pressure, intensive smartphone use, and limited offline social opportunities has been described as a “perfect storm” for the accumulation of stress (Park & Park, 2022). On this account, the principal pathway from problematic smartphone use to broad domains of adolescent well-being runs through stress. This expectation is grounded in prospective evidence: academic stress forecasts subsequent depression and diminished well-being in Korean adolescents (Lee & Lee, 2021), and academic stress and smartphone dependency reinforce one another across time, with stress operating as both an antecedent and a consequence of overdependence (Park & Park, 2022). The transactional model further implies that this stress process should generalize across affective and relational domains rather than remaining confined to a single outcome, motivating the prediction that academic stress mediates associations with school adjustment, ego resilience, and subjective happiness alike.

The second is a time-displacement mechanism. Time spent on smartphones, particularly in passive consumption modes such as gaming and short-form video, competes for finite time and attentional resources with effortful activities such as self-directed studying (Neuman, 1988; Vanden Abeele et al., 2022). Because adolescent brains undergo asymmetric maturation in reward-seeking versus cognitive-control systems (Casey et al., 2008; Steinberg, 2008), high-reward-salience screen activities preferentially capture attention and time, displacing investment in learning. Empirical support for this displacement account comes from studies showing that problematic gaming, heavy social media use, and media-induced task switching during studying are associated with poorer school performance and learning-related outcomes (Gentile et al., 2011; Rosen et al., 2013; Sampasa-Kanyinga et al., 2019; George et al., 2020). This mechanism is expected to selectively undermine learning-relevant outcomes, particularly self-regulated learning, while exerting weaker effects on broader well-being indicators. The hypothesis rests on prospective evidence that pathological gaming predicts later academic and psychosocial difficulties (Gentile et al., 2011) and that daily digital-technology use is prospectively associated with adolescent functioning (George et al., 2020). Because self-regulated learning depends on the sustained allocation of the very time and attentional resources that high-reward screen activities consume, the displacement account predicts a domain-specific rather than a diffuse effect: gaming and reduced self-study should mediate associations with self-regulated learning, whereas affective outcomes should be comparatively spared.

Importantly, prior research has tended to examine these mechanisms in isolation: studies grounded in stress frameworks emphasize emotional dysregulation and psychosocial strain, whereas time-displacement studies focus on behavioral trade-offs in time use, and the two have rarely been tested simultaneously within a unified longitudinal framework. However, the mechanisms are not mutually exclusive—a single adolescent may experience both elevated stress and altered time allocation, and different developmental outcomes may be differentially associated with each. A dual-mechanism model that tests both processes simultaneously may therefore better capture the developmental impact of problematic smartphone use than models that assume a single underlying pathway.

### 1.3. Limitations of Prior Longitudinal Research

Despite the maturity of cross-sectional findings, three notable limitations characterize the existing longitudinal literature on problematic smartphone use and adolescent development. First, most existing work focuses on a single outcome rather than examining a portfolio of developmental indicators that together capture the school, regulatory, resilience, and well-being domains. Second, mediating mechanisms are often theorized but rarely formally tested with longitudinal designs that incorporate temporally ordered a- and b-paths while controlling for baseline stability. Third, the few longitudinal mediation studies that do exist tend to treat stress and time displacement as competing explanations, rather than as potentially complementary processes.

Critically, these limitations have prevented the field from determining whether problematic smartphone use operates as a general developmental risk factor or as a constellation of domain-specific influences. Addressing this question requires a design that simultaneously models multiple mediators and multiple outcomes across time.

A further limitation is the absence of integrative designs that can compare mediation patterns across outcomes. A multi-outcome, multi-mediator framework is necessary to determine whether mechanisms generalize or remain outcome-specific. The present study addresses this gap by simultaneously examining four developmental outcomes and four mediators, yielding 16 theoretically specified indirect pathways. This design enables direct comparison of mediation profiles across domains within a single analytic framework.

### 1.4. The Present Study

Using three consecutive waves (W14–W16) of the Panel Study on Korean Children (PSKC), this study examined whether problematic smartphone use at age 14 predicts four developmental outcomes at age 16 and whether these longitudinal associations are mediated by age-15 gaming hours, video-watching hours, self-study hours, and academic stress.

**Figure 1:**
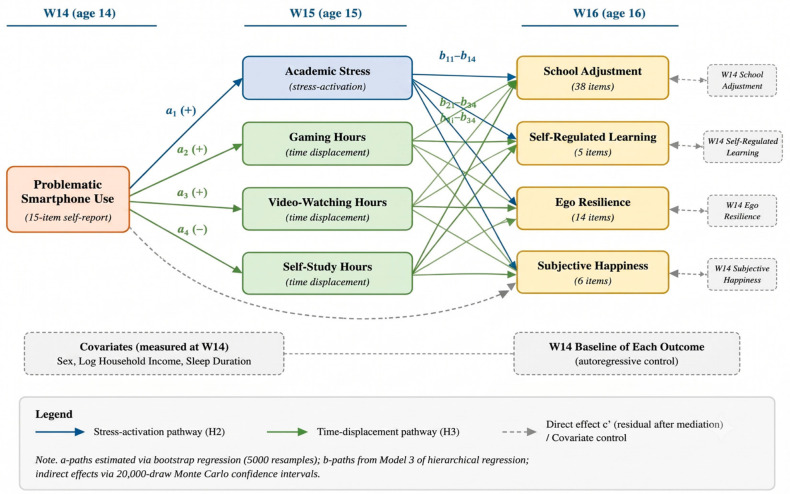
Conceptual dual-mechanism mediation model. Problematic smartphone use at W14 (T7) is hypothesized to predict four developmental outcomes at W16 (T9) through two parallel mediating mechanisms operating at W15 (T8): a stress-activation pathway (blue arrows) operating through academic stress, and a time-displacement pathway (green arrows) operating through gaming hours, video-watching hours, and reduced self-study hours. The dashed gray arrow represents the residual direct effect (c′). Each outcome regression includes its own W14 autoregressive baseline and three covariates (sex, log income, and sleep hours).

The four outcomes were deliberately selected to span four conceptually distinct developmental domains—school-related, regulatory, resilience, and emotional—so that the design could test whether problematic smartphone use functions as a general developmental risk factor or exerts domain-specific influence. School adjustment indexes adolescents’ behavioral and relational functioning within the school context. Self-regulated learning (Zimmerman, 2008) reflects the metacognitive and motivational processes involved in managing one’s own learning and was included as the outcome most theoretically proximal to the time-displacement mechanism. Ego resilience (Block & Kremen, 1996) refers to the capacity to regulate ego control adaptively in response to environmental demands and indexes emotional-regulatory functioning that is expected to be sensitive to stress mechanisms. Subjective happiness (Diener & Seligman, 2002) represents a global affective evaluation of one’s life and was included as a broad well-being indicator against which the specificity of the two pathways could be evaluated. The conceptual model is presented in Figure 1.

The four mediators were chosen to operationalize the two theorized mechanisms as directly as possible. Academic stress served as the affective indicator of the stress-activation mechanism, capturing the perceived demand–resource imbalance through which problematic smartphone use is hypothesized to erode well-being. Gaming hours, video-watching hours, and self-study hours served as behavioral indicators of the time-displacement mechanism: gaming and video-watching represent the two most prevalent high-engagement screen activities that compete for adolescents’ finite time, whereas self-study hours index the effortful, learning-relevant activity expected to be displaced. Gaming and video-watching were modeled separately rather than as a single screen-time index because they differ in interactivity and reward structure and may therefore relate differently to learning and developmental outcomes (Gentile et al., 2011; Vanden Abeele et al., 2022). Together, the mediator set maps one affective and three behavioral pathways onto the dual-mechanism model, allowing the stress and time-displacement processes to be tested simultaneously within a single design.

Hierarchical regression analyses with autoregressive controls quantify each outcome’s susceptibility to problematic smartphone use beyond baseline functioning. Bootstrap regressions establish a-paths from problematic smartphone use to each W15 mediator. Monte Carlo confidence intervals (MacKinnon et al., 2020; Selig & Preacher, 2008; Tofighi & MacKinnon, 2016) assess the statistical significance of all 16 indirect effects (4 mediators × 4 outcomes), with Benjamini–Hochberg false discovery rate correction applied as a sensitivity analysis to address concerns about multiple inference.

Based on the dual-mechanism framework, the following hypotheses were theoretically specified. Because the study was not preregistered, these hypotheses should be interpreted as theory-driven expectations rather than confirmatory preregistered predictions.

**H1.**  *Higher levels of problematic smartphone use at W14 will be associated with less favorable developmental outcomes at W16 (school adjustment, self-regulated learning, ego resilience, and subjective happiness), controlling for each outcome’s autoregressive baseline and relevant covariates.*

**H2.**  *Indirect associations through academic stress (problematic smartphone use → academic stress → outcome) are expected to be observed for school adjustment, ego resilience, and subjective happiness, consistent with a stress-activation pathway.*

**H3.**  *Indirect associations through gaming hours and reduced self-study hours (problematic smartphone use → mediator → outcome) are expected to be observed for self-regulated learning, consistent with a time-displacement pathway.*

## 2. Methods

### 2.1. Data Source and Participants

Data were drawn from the Panel Study on Korean Children (PSKC), a nationally representative longitudinal birth-cohort panel administered by the Korea Institute of Child Care and Education (2025). The PSKC began in 2008 with 2150 households and has followed the cohort annually, collecting data from children, parents, caregivers, teachers, and schools.

The present study used three consecutive waves, namely Wave 14 (2021; age 14), Wave 15 (2022; age 15), and Wave 16 (2023; age 16), corresponding to the middle school years. By Wave 16, 1508 households (70.1% of the original sample) remained.

Analyses were based on adolescent self-report data, with sample sizes varying slightly across models due to missing data. The bootstrap a-path analyses used all cases with available data on problematic smartphone use and the corresponding mediator (N = 1116). In contrast, the hierarchical regression models additionally required complete data on outcome variables and covariates, resulting in slightly smaller analytic samples (N = 1068–1072). The Wave 14 sample was 51.0% boys and 49.0% girls.

Attrition analyses comparing participants retained through Wave 16 with those lost to follow-up indicated no significant differences in sex, household income, or sleep duration at baseline. However, adolescents who were later lost to follow-up reported slightly higher levels of academic stress and problematic smartphone use at baseline. Although these differences were modest, selective attrition cannot be ruled out and should be considered when interpreting the findings.

The PSKC obtained informed parental consent and adolescent assent at each wave. The present study used de-identified secondary data and was exempt from additional ethical review.

### 2.2. Measures

#### 2.2.1. Problematic Smartphone Use (W14)

Problematic smartphone use was assessed at W14 with the 15-item Korean Smartphone Addiction Proneness Scale for Youth, developed by the National Information Society Agency (2011) and validated in youth samples (D. Kim et al., 2014). Items were rated on a 4-point scale (1 = strongly disagree, 4 = strongly agree), with Items 8, 10, and 13 reverse-scored prior to averaging; higher scores indicate greater problematic smartphone use. The scale is a non-diagnostic, self-report measure of overdependence with no validated clinical cut-off; scores should therefore not be interpreted as indicating a clinical addiction diagnosis. Internal consistency was acceptable (Cronbach’s α = 0.856). The mean score was 2.00 (SD = 0.43), with skewness = 0.04 and kurtosis = 0.14, indicating an approximately normal distribution within the four-point range.

#### 2.2.2. W15 Behavioral and Affective Mediators

Four W15 mediators were assessed by adolescent self-report covering the past week. Gaming hours (M = 0.80, SD = 0.96, range 0–8.5), video-watching hours (M = 1.29, SD = 0.95, range 0–7.5), and self-study hours (M = 1.02, SD = 0.85, range 0–5.0) are reported in average daily hours. Academic stress was assessed using four adolescent self-report items from the Panel Study on Korean Children (PSKC) questionnaire, covering perceived burden related to studying, examinations, and parental and teacher expectations. Responses were recorded on a 5-point Likert scale ranging from 1 (never) to 5 (always), and mean scores were calculated; higher values indicated greater academic stress (M = 2.67, SD = 0.88; Cronbach’s α = 0.826).

Gaming hours and video-watching hours were analyzed as separate mediators because these activities differ conceptually in their degree of interactivity and cognitive engagement. Consistent with prior media-use research (Gentile et al., 2011; Vanden Abeele et al., 2022), gaming typically involves active participation and reward-based feedback processes, whereas video watching is generally more passive and consumption-oriented. These differences suggest that the two forms of screen use may show distinct associations with learning and developmental outcomes. Therefore, they were modeled separately rather than combined into a single recreational screen-time variable.

#### 2.2.3. W16 Developmental Outcomes

Four developmental outcomes were assessed at W16 with self-report instruments. School adjustment (38 items, 5-point scale; Cronbach’s α = 0.942; W14 baseline M = 3.86, W16 outcome M = 3.80) measured the adolescent’s relational, behavioral, and motivational fit with school. Self-regulated learning (Zimmerman, 2008; 5 items, 4-point scale; α = 0.809; W14 M = 2.61, W16 M = 2.68) captured metacognitive planning, monitoring, and effort regulation in learning. Ego resilience (Block & Kremen, 1996; 14 items, 4-point scale; α = 0.833; W14 M = 2.91, W16 M = 2.82) indexed adaptive ego modulation. Subjective happiness (6 items, 4-point scale; α = 0.746; W14 M = 2.93, W16 M = 2.82) was a global self-evaluation of happiness. The W14 baseline of each outcome was used as the autoregressive control in the corresponding regression model.

#### 2.2.4. Covariates

Three covariates were included in all regression models: sex (0 = male, 1 = female), log-transformed household income at W14 (M = 6.30, SD = 0.52), and weekday sleep duration at W14 (M = 7.46, SD = 1.28). These variables were included because prior research has consistently linked demographic characteristics, socioeconomic status, and sleep patterns to both problematic smartphone use and adolescent developmental outcomes, making them plausible confounders of the associations examined in this study.

For the regression analyses, all continuous variables were standardized (z-scores) to facilitate comparison of effect sizes across predictors and outcomes. The binary sex variable was retained in dummy-coded form and was not standardized. Consequently, regression coefficients for continuous predictors represent standardized effects, whereas the coefficient for sex reflects the mean difference between females and males, adjusted for other variables in the model.

### 2.3. Analytic Strategy

#### 2.3.1. Hierarchical Regression

Four parallel hierarchical regression models were estimated, one for each Wave 16 outcome. In Step 1, the autoregressive baseline (Wave 14 measure of the same outcome) and covariates (sex, household income, and sleep duration) were entered. In Step 2, problematic smartphone use at Wave 14 was added. In Step 3, the Wave 15 mediators (gaming hours, video-watching hours, self-study hours, and academic stress) were included. Changes in explained variance (Δ*R*^2^) and corresponding F-change statistics were examined at each step. Multicollinearity was assessed using variance inflation factors (VIFs) and tolerance values, computed from the full Step-3 model and therefore including the four mediators.

#### 2.3.2. Bootstrap A-Path Estimation

Bootstrap regression analyses were conducted to estimate the a-paths from problematic smartphone use in Wave 14 to each Wave 15 mediator. Four separate models were estimated, with each mediator (gaming hours, video-watching hours, self-study hours, and academic stress) specified as the dependent variable and problematic smartphone use entered as the predictor, controlling for sex, household income, and sleep duration. Percentile 95% confidence intervals were obtained using 5000 bootstrap resamples for all four models, consistent with Hayes’s (2018) recommendation; harmonizing the resample count (from the previous 5000/2000 mix) did not change the significance or substantive magnitude of any a-path (all |Δβ| ≤ 0.003).

#### 2.3.3. Indirect Effects and Monte Carlo Confidence Interval Procedure

Sixteen indirect effects (4 mediators × 4 outcomes) were computed as the product of the a-path and b-path coefficients. Consistent with contemporary mediation practice, indirect effects were evaluated directly rather than being conditioned on a significant total effect (Zhao et al., 2010). The a-paths were obtained from the bootstrap regression models described above, and the b-paths were taken from Step 3 of the corresponding hierarchical regression models.

95% Monte Carlo confidence intervals (MCIs) were constructed for each indirect effect using 20,000 simulated draws based on the point estimates and standard errors of the a- and b-paths, following established procedures (Selig & Preacher, 2008; Tofighi & MacKinnon, 2016). An indirect effect was considered statistically significant if the 95% MCCI did not include zero.

Given the multiple tests (16 indirect effects), the Benjamini–Hochberg false discovery rate (FDR) procedure was applied at q = 0.05 as a sensitivity analysis (Benjamini & Hochberg, 1995). Both unadjusted and FDR-adjusted results are reported in in the Section 3.

#### 2.3.4. Software and Reproducibility

All statistical analyses were conducted using IBM SPSS Statistics (Version 30) and Python 3.10. Bootstrap procedures and false discovery rate corrections were applied where appropriate. Analytical procedures are available from the author upon reasonable request.

## 3. Results

### 3.1. Preliminary Analyses

Descriptive statistics indicated that all study variables were within acceptable ranges of normality (absolute skewness ≤ 2.0; kurtosis ≤ 7.0). Zero-order correlations showed that problematic smartphone use at Wave 14 was negatively associated with all four developmental outcomes at Wave 16, with coefficients ranging from *r* = −0.178 (ego resilience) to *r* = −0.227 (school adjustment), all *p* < 0.001. Across all final regression models, variance inflation factors ranged from 1.03 to 1.32, indicating no concerns regarding multicollinearity. These VIF values were computed from the full Step-3 model and therefore include the four mediators (gaming, video, self-study, and academic stress) alongside problematic smartphone use and the covariates; no pair, including gaming and self-study hours, approached problematic collinearity.

### 3.2. Hierarchical Regression Models

Four hierarchical regression models were estimated to examine the longitudinal associations between Wave 14 problematic smartphone use and each Wave 16 developmental outcome, controlling for autoregressive baseline levels and covariates (sex, log-transformed household income at Wave 14, and sleep duration at Wave 14).

In Step 1, covariates and baseline measures were entered; in Step 2, problematic smartphone use was added to assess its incremental contribution; and in Step 3, the Wave 15 mediators (gaming hours, video-watching hours, self-study hours, and academic stress) were included to examine attenuation of the association between problematic smartphone use and each outcome. Model fit statistics are reported in Table 1, and standardized coefficients from the full models (Step 3) are presented in Table 2.

Step 1 was statistically significant across all models, with R^2^ ranging from 0.176 (self-regulated learning) to 0.259 (subjective happiness).

In Step 2, adding problematic smartphone use accounted for additional variance in school adjustment (Δ*R*^2^ = 0.005, *p* = 0.005), self-regulated learning (Δ*R*^2^ = 0.013, *p* < 0.001), and ego resilience (Δ*R*^2^ = 0.006, *p* = 0.003), but not in subjective happiness *(*Δ*R*^2^ = 0.001, *p* = 0.161). The corresponding Step-2 standardized total-effect coefficients of problematic smartphone use were *β* = −0.078 (*p* = 0.005) for school adjustment, *β* = −0.118 (*p* < 0.001) for self-regulated learning, *β* = −0.082 (*p* = 0.003) for ego resilience, and *β* = −0.039 (*p* = 0.161, n.s.) for subjective happiness. Thus, H1 was supported for three of the four outcomes; the association with subjective happiness was indirect only.

In Step 3, the inclusion of the Wave 15 mediators significantly increased explained variance for all outcomes, with the largest increase observed for subjective happiness (Δ*R*^2^ = 0.040). Among the mediators, academic stress showed the strongest association with subjective happiness (*β* = −0.201, *p* < 0.001).

The direct effect of problematic smartphone use in Step 3 was no longer statistically significant for school adjustment (*β* = −0.045, *p* = 0.109) and ego resilience *(β* = −0.055, *p* = 0.054) and remained non-significant for subjective happiness *(β* = 0.001, *p* = 0.977). In contrast, self-regulated learning retained a significant direct association (*β* = −0.087, *p* = 0.003).

This attenuation pattern is consistent with the possibility that the observed longitudinal associations operate, at least in part, through the proposed mediators. However, because mediation cannot be definitively established in observational panel data, subsequent indirect-effect analyses should be interpreted as evidence of prospective associations consistent with the hypothesized pathways rather than as definitive causal mechanisms.

### 3.3. Bootstrap A-Paths from Smartphone Addiction to W15 Mediators

All four a-paths were statistically significant, with bootstrap 95% confidence intervals excluding zero (Table 3). Problematic smartphone use at W14 was associated with higher gaming hours (*β* = 0.119, 95% CI [0.057, 0.185]), higher video-watching hours (*β* = 0.127, 95% CI [0.075, 0.188]), and higher academic stress (*β* = 0.172, 95% CI [0.113, 0.234]), and with lower self-study hours (*β* = −0.146, 95% CI [−0.212, −0.090]).

Among the four mediators, the largest unadjusted association was observed for academic stress. However, because these estimates do not account for baseline levels of the mediators, their interpretation should be considered provisional pending the sensitivity analyses reported below.

### 3.4. Indirect Effects via Monte Carlo Confidence Intervals

**Figure 2:**
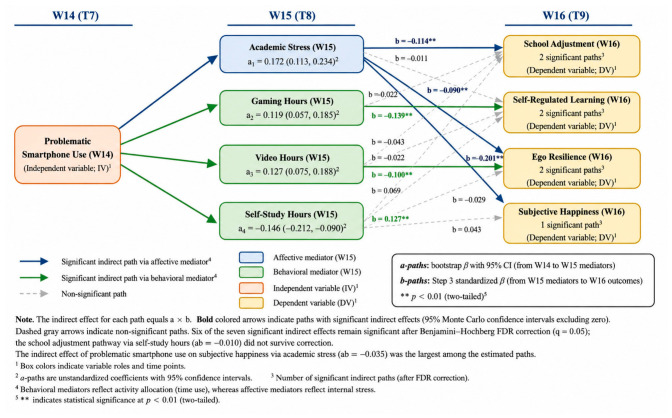
Statistical model with significant indirect pathways highlighted. Bold colored arrows indicate paths with significant indirect effects (a × b; 95% Monte Carlo confidence intervals excluding zero). Dashed gray arrows indicate non-significant paths. Coefficients are standardized; a-paths show bootstrap *β* (95% CI), and b-paths show standardized β from Step 3 hierarchical regression models. Six of the seven significant indirect effects remained significant after Benjamini–Hochberg false discovery rate correction (*q* = 0.05); the school adjustment pathway via self-study hours (ab = −0.010) did not survive correction. The indirect effect of problematic smartphone use on subjective happiness via academic stress (ab = −0.035) was the largest among the estimated paths.

Of the 16 indirect effects, 7 were statistically significant under unadjusted Monte Carlo confidence intervals (95% MCCIs excluded zero), and all 7 were negative (Table 4). Under the more stringent Benjamini–Hochberg false discovery rate correction (*q* = 0.05), 6 of the 7 paths survived; the only path that became non-significant under BH-FDR was the school adjustment pathway through self-study hours (ab = −0.0101, unadjusted *p* = 0.043). The pattern of significant indirect effects is summarized graphically in Figure 2.

Three patterns warrant emphasis. First, academic stress was a significant mediator for three of the four outcomes, namely school adjustment (ab = −0.020, 95% MCCI [−0.032, −0.009]), ego resilience (ab = −0.016, [−0.027, −0.006]), and subjective happiness (ab = −0.035, [−0.051, −0.021]), all of which survived BH-FDR correction. Under the primary analytic specification, academic stress emerged as the most pervasive mediator, showing significant indirect associations with school adjustment, ego resilience, and subjective happiness. However, as shown in the sensitivity analyses, these stress-mediated pathways were not robust to adjustment for baseline levels of academic stress and should therefore be interpreted with caution. Second, under the primary analytic specification, the largest indirect effect was observed for subjective happiness through academic stress (ab = −0.035). However, as noted below, this pathway was not robust to adjustment for baseline levels of academic stress and should therefore be interpreted cautiously.

Third, self-regulated learning displayed a distinct pattern: significant indirect effects operated through gaming hours (ab = −0.017) and self-study hours (ab = −0.019), but not stress, with both paths surviving BH-FDR correction (H3 supported). Ego resilience also showed an additional indirect effect through video hours that survived BH-FDR correction.

### 3.5. Sensitivity and Robustness Analyses

To address the possibility that the a-paths reflected pre-existing differences rather than prospective change, all four a-paths were re-estimated, controlling for the Wave-14 baseline level of each mediator. The three time-use a-paths remained significant after baseline adjustment (gaming *β* = 0.056, *p* = 0.031; video *β* = 0.082, *p* = 0.004; self-study *β* = −0.098, *p* < 0.001), indicating that problematic smartphone use prospectively predicts change in these behaviors. In contrast, the academic-stress a-path was fully attenuated (*β* = 0.172 → 0.002, *p* = 0.94). When the indirect effects were re-estimated under baseline adjustment, the time-use pathways to self-regulated learning and ego resilience remained significant (two surviving false discovery rate corrections). In contrast, none of the academic-stress–mediated indirect effects were significant. These findings materially qualify the interpretation of the primary mediation analyses. The time-use pathways remained evident after adjustment for baseline mediator levels, indicating prospective associations between problematic smartphone use and subsequent changes in gaming, video-watching, and self-study behaviors. In contrast, the academic-stress pathway was fully attenuated after baseline adjustment, suggesting that the stress-related associations observed in the primary analyses primarily reflect stable between-person differences already present at baseline rather than prospective within-person change.

The full model was additionally re-estimated as an integrated path model in which all four mediators and four outcomes were estimated simultaneously, allowing the mediators and the outcome residuals to covary; model fit was good (CFI = 0.970, TLI = 0.940, RMSEA = 0.043), and the same seven indirect effects were significant, with the academic-stress-to-subjective-happiness path again the largest. The pattern was unchanged under full-information maximum likelihood (FIML) estimation using all available cases (N = 1395) and under inverse-probability weighting using the PSKC longitudinal panel weight, indicating that the findings are not an artifact of listwise deletion or differential attrition.

## 4. Discussion

Using three waves of the Panel Study on Korean Children, this study found that problematic smartphone use at age 14 was associated with developmental outcomes at age 16. The observed associations were consistent with two theoretically specified pathways—academic stress and time displacement—although subsequent robustness analyses provided stronger support for the time-displacement pathway than for the stress pathway.

Of the 16 hypothesized indirect paths, 7 were statistically significant based on unadjusted Monte Carlo confidence intervals, and 6 remained significant after Benjamini–Hochberg false discovery rate correction (*q* = 0.05). All significant effects were in the hypothesized direction.

The overall pattern of findings was consistent with the theoretically specified dual-mechanism framework, with differential mediation profiles observed across outcomes. Accordingly, interpretation focuses on the convergence of stress-mediated and time-displacement-mediated pathways rather than on any single indirect effect.

### 4.1. Academic Stress as a Concurrent Correlate of Developmental Vulnerability

Under the primary analytic specification, academic stress emerged as a significant indirect pathway linking problematic smartphone use to school adjustment, ego resilience, and subjective happiness. The indirect effect through academic stress on subjective happiness was the largest among the estimated pathways and remained significant after false discovery rate correction. However, these findings were not robust to adjustment for baseline levels of academic stress. When Wave-14 academic stress was included as a control, the association between problematic smartphone use and Wave-15 academic stress was fully attenuated (see Section 3.5). This pattern suggests that adolescents with higher levels of problematic smartphone use already exhibited elevated academic stress at baseline, indicating a co-occurring vulnerability profile rather than a prospective increase in stress attributable to problematic smartphone use.

This interpretation is consistent with the transactional stress model (Lazarus & Folkman, 1984), which conceptualizes stress as arising when perceived demands exceed available coping resources. Problematic smartphone use may be associated with disruptions to sleep, study routines, and offline engagement, all of which are themselves linked to elevated academic stress and poorer developmental outcomes.

This between-person interpretation aligns with recent longitudinal work published in this journal. Examining problematic smartphone use and materialism across three annual waves, Wang (2026) found reciprocal associations at the between-person level but no significant cross-lagged effects within persons, illustrating how readily unadjusted or cross-sectional mediation estimates can capture stable trait-like differences rather than genuine prospective change. The present results extend this caution to the academic-stress pathway. Although problematic smartphone use and academic stress co-occur, the baseline-adjusted analyses indicate that this co-occurrence reflects pre-existing differences between adolescents rather than a process by which smartphone use prospectively raises stress. The distinction has practical weight because cross-sectional studies in this journal have reported that mental stress mediates the link between problematic smartphone use and lowered self-efficacy (Feng, 2024); our longitudinal findings suggest that such stress-mediation estimates may be inflated when baseline stress is left uncontrolled and that the apparent breadth of the stress pathway is better read as a marker of co-occurring vulnerability than as a modifiable mechanism.

Notably, problematic smartphone use was not directly associated with subjective happiness; the association emerged only indirectly, through academic stress, under the primary analytic specification.

### 4.2. Time Displacement as a Domain-Specific Mediator

Self-regulated learning showed a distinct mediation profile, supporting H3. Academic stress was not a significant mediator of the association between problematic smartphone use and self-regulated learning. In contrast, gaming hours and reduced self-study hours were, and both pathways remained significant after false discovery rate correction. This dissociation is theoretically informative. Whereas the affective outcomes were linked to problematic smartphone use through perceived stress, self-regulated learning—a behavior that depends on the concrete allocation of study time—was linked through the reallocation of time itself. The mediator that mattered for learning was thus the displaced behavior rather than the emotional appraisal, indicating that the time-displacement mechanism operates in a domain-specific rather than a diffuse manner and that learning outcomes are sensitive to where adolescents’ finite time is spent, not merely to how stressed they feel.

This pattern is consistent with the time-displacement perspective (Rosen et al., 2013; Hartanto et al., 2021; Gentile et al., 2011), which emphasizes the allocation of time and attention. Self-regulated learning depends on sustained investment of these resources, and smartphone-related activities may be associated with shifts toward gaming and away from self-study. In this sense, the mechanism appears more behavioral than affective.

Self-regulated learning was also the only outcome that retained a significant direct association with problematic smartphone use (*β* = −0.087, *p* = 0.003). This suggests that additional processes not captured by the current mediators may be involved, such as attentional disruption, sleep-related factors, or motivational changes. Future research could directly test these possibilities by incorporating objective measures of attentional control, executive functioning, and task-switching behavior, as well as digital trace data capturing real-time smartphone use patterns. Experimental and intensive longitudinal designs may help clarify whether attentional disruption represents an independent pathway linking problematic smartphone use to self-regulated learning.

### 4.3. Convergent Evidence for a Dual-Mechanism Model

Taken together, the findings are broadly consistent with a dual-mechanism account. However, the robustness analyses suggest that the evidence is stronger for the time-displacement pathway than for the stress pathway, which was attenuated after adjustment for baseline academic stress.

Problematic smartphone use in early adolescence appears to be associated with two related patterns. First, adolescents reporting higher levels of problematic smartphone use also reported higher levels of academic stress, which were associated with poorer well-being, school adjustment, and emotional resilience. Second, problematic smartphone use was prospectively associated with shifts in time use toward media activities and away from self-directed learning, which were more closely related to self-regulated learning outcomes.

These processes are not mutually exclusive and may co-occur within the same individual. For example, increased gaming time may be associated with both reduced study time and elevated stress. Overall, the findings suggest that no single mechanism fully accounts for the associations between problematic smartphone use and adolescent development. The strongest evidence was observed for time-allocation processes as prospective pathways. In contrast, stress-related processes appeared to characterize a broader vulnerability profile that co-occurred with higher levels of problematic smartphone use. Interventions may therefore benefit from addressing both domains while recognizing that the empirical support for the time-displacement pathway is currently stronger.

### 4.4. Practical Implications

These findings may inform hypotheses for future school-based digital well-being interventions in Korea and similar contexts.

First, academic stress was associated with poorer adjustment and well-being outcomes and may represent an important marker of developmental vulnerability among adolescents with higher levels of problematic smartphone use. Accordingly, future intervention studies may examine whether programs targeting stress reduction are associated with improved outcomes among adolescents with higher levels of problematic smartphone use.

Second, self-regulated learning appears to be particularly sensitive to time-displacement processes and retains a direct association with problematic smartphone use. This suggests that future studies may evaluate whether study skill interventions and time-management training function as useful complements to digital-hygiene programs.

Third, the relatively small total association between problematic smartphone use and subjective happiness, coupled with a significant indirect pathway through stress in the primary analyses, suggests caution in framing messages that focus solely on screen time. Rather, these findings indicate that the stress-related context of smartphone use may be more relevant to adolescent well-being than screen exposure alone.

### 4.5. Limitations and Future Directions

Several limitations qualify these conclusions. All constructs were assessed by self-report, raising the possibility of common-method and reporting-style variance—a concern most acute for the affective academic stress and subjective happiness measures, which reinforces a cautious, associational reading of the stress pathway. The mediators examined do not exhaust the relevant processes; sleep, family context, peer relationships, and unmeasured confounders such as prior mental health, parental monitoring, and academic achievement may contribute residual confounding, so the coefficients are best read as adjusted associations rather than causal effects. The estimates derive from regression rather than a latent-variable framework, leaving measurement error unmodeled. Finally, the sample is a single Korean cohort whose Wave-14 data were collected in 2021 under COVID-19 disruptions that may have jointly elevated screen time and stress. However, the baseline-controlled analyses (Section 3.5) partially address such period effects, and FIML and inverse-probability weighting indicated that the modestly higher-risk attrition rendered the estimates conservative rather than biased; generalization beyond this context warrants caution, and because not all indirect effects survived correction for multiple comparisons, replication in independent samples is needed.

These limitations point to clear next steps. Future work should incorporate learning-specific mediators—particularly objective indices of attentional control, executive functioning, and task-switching—alongside digital-trace measures of real-time use to identify the processes underlying the residual direct effect on self-regulated learning. Cross-lagged panel SEM across additional waves, extending follow-up into later adolescence and emerging adulthood, would clarify whether the observed pathways are reciprocal and whether they persist or change over time. Finally, intervention studies that directly contrast stress-regulation with time-management approaches are needed to establish whether the prospective time-displacement pathway is causally modifiable.

## 5. Conclusions

Problematic smartphone use in early adolescence was prospectively associated with later adolescent functioning, broadly consistent with a dual-pathway framework involving academic stress and time use. Of the 16 indirect effects examined, 7 were statistically significant under unadjusted criteria, and 6 remained significant after false discovery rate correction.

Under the primary analytic specification, academic stress emerged as the most pervasive indirect pathway, linking problematic smartphone use to school adjustment, ego resilience, and subjective happiness. However, these associations were not robust to adjustment for baseline levels of academic stress and therefore appear to reflect a broader vulnerability profile rather than a prospective stress-mediated process. In contrast, the time-use pathways involving increased gaming and reduced self-study remained significant after baseline adjustment, providing stronger evidence for prospective associations with self-regulated learning outcomes.

Taken together, and given the modest magnitude of the observed associations, these findings generate hypotheses for future intervention research focused on both stress-related vulnerabilities and time-management processes rather than providing direct intervention prescriptions. These findings contribute longitudinal evidence that the developmental correlates of problematic smartphone use are better understood as outcome-specific processes operating through distinct mechanisms rather than through a single generalized pathway.

## Figures and Tables

**Table 1:** Hierarchical regression model summary across four developmental outcomes.

Outcome	Model	*R*	*R* ^2^	Adj. *R*^2^	Δ*R*^2^	F Change	df1, df2	*p*
School Adjustment	1	0.508	0.258	0.255	0.258	92.35	4, 1063	<0.001
	2	0.513	0.263	0.260	0.005	7.89	1, 1062	0.005
	3	0.530	0.281	0.275	0.018	6.62	4, 1058	<0.001
Self-Regulated Learning	1	0.419	0.176	0.173	0.176	56.89	4, 1067	<0.001
	2	0.434	0.189	0.185	0.013	17.01	1, 1066	<0.001
	3	0.476	0.226	0.220	0.038	12.94	4, 1062	<0.001
Ego Resilience	1	0.478	0.228	0.225	0.228	78.89	4, 1067	<0.001
	2	0.484	0.235	0.231	0.006	8.87	1, 1066	0.003
	3	0.500	0.250	0.244	0.015	5.44	4, 1062	<0.001
Subjective Happiness	1	0.509	0.259	0.256	0.259	93.12	4, 1067	<0.001
	2	0.510	0.260	0.257	0.001	1.96	1, 1066	0.161
	3	0.548	0.300	0.294	0.040	15.16	4, 1062	<0.001

**Table 2:** Standardized regression coefficients for the final hierarchical model (Model 3) predicting four developmental outcomes at W16.

Predictor	School Adjustment	Self-Regulated Learning	Ego Resilience	Subjective Happiness
	*β* (*SE*)	*β* (*SE*)	*β* (*SE*)	*β* (*SE*)
**Step 1: Covariates**				
Female	0.010 (0.029)	0.067 * (0.030)	−0.073 * (0.029)	−0.097 ** (0.031)
Log income	0.015 (0.029)	0.054 * (0.030)	0.004 (0.029)	0.068 * (0.028)
Sleep duration	−0.031 (0.029)	−0.064 * (0.030)	−0.042 (0.029)	−0.067 * (0.028)
Autoregressive baseline (Prior-wave outcome)	0.458 *** (0.028)	0.285 *** (0.029)	0.435 *** (0.028)	0.447 *** (0.027)
**PSU (Direct Effect, Step 3)**				
PSU	−0.045 (0.029)	−0.087 ** (0.029)	−0.055 (0.028)	0.001 (0.028)
**Step 3: Mediators**				
Gaming	−0.022 (0.029)	−0.139 *** (0.030)	−0.036 (0.029)	−0.052 (0.028)
Video	−0.043 (0.028)	−0.022 (0.029)	−0.100 *** (0.028)	−0.029 (0.027)
Study	0.069 * (0.030)	0.127 *** (0.031)	−0.018 (0.030)	0.043 (0.029)
Stress	−0.114 *** (0.027)	−0.011 (0.028)	−0.090 ** (0.027)	−0.201 *** (0.026)

Note. All continuous variables were standardized prior to analysis. The sex variable was retained in dummy-coded form (0 = male, 1 = female). *β* = standardized coefficient for continuous predictors. SE = standard error. PSU = problematic smartphone use. The coefficients in the “PSU (Direct Effect, Step 3)” section represent the direct effects of problematic smartphone use after the four Wave-15 mediators were entered into the final model. The corresponding Step-2 total-effect coefficients are reported in the text. The autoregressive baseline row reports the standardized coefficient of the prior-wave (W14) measure of the corresponding outcome. * *p* < 0.05; ** *p* < 0.01; *** *p* < 0.001.

**Table 3:** Bootstrap regression of W14 problematic smartphone use predicting W15 mediators (a-paths).

Mediator	*β*	SE	*p*	95% LL	95% UL	*R* ^2^
Gaming hours	0.119	0.032	<0.001	0.057	0.185	0.157
Video hours	0.127	0.029	<0.001	0.075	0.188	0.089
Self-study hours	−0.146	0.031	<0.001	−0.212	−0.090	0.124
Academic Stress	0.172	0.031	<0.001	0.113	0.234	0.047

Note. Each row reports a separate bootstrap regression in which the W15 mediator was specified as the outcome, controlling for sex, log-transformed household income, and sleep duration. All variables were standardized. Percentile 95% confidence intervals were obtained from 5000 bootstrap resamples for all four models. N = 1116.

**Table 4:** Indirect effects of W14 problematic smartphone use on W16 outcomes through W15 mediators (16 paths).

Outcome	Mediator	a	b	a × b	95% LL	95% UL
School Adjustment	Gaming	0.119	−0.022	−0.003	−0.011	0.004
	Video	0.127	−0.043	−0.006	−0.014	0.002
	Self-Study	−0.146	0.069	−0.010 *	−0.021	−0.001
	Academic Stress	0.172	−0.114	−0.020 **	−0.032	−0.009
Self-regulated Learning	Gaming	0.119	−0.139	−0.017 **	−0.029	−0.007
	Video	0.127	−0.022	−0.003	−0.011	0.004
	Self-Study	−0.146	0.127	−0.019 **	−0.032	−0.008
	Academic Stress	0.172	−0.011	−0.002	−0.012	0.008
Ego Resilience	Gaming	0.119	−0.036	−0.004	−0.013	0.003
	Video	0.127	−0.100	−0.013 **	−0.023	−0.005
	Self-Study	−0.146	−0.018	0.003	−0.006	0.012
	Academic Stress	0.172	−0.090	−0.016 **	−0.027	−0.006
Subjective Happiness	Gaming	0.119	−0.052	−0.006	−0.015	0.000
	Video	0.127	−0.029	−0.004	−0.011	0.003
	Self-Study	−0.146	0.043	−0.006	−0.016	0.002
	Academic Stress	0.172	−0.201	−0.035 **	−0.051	−0.021

Note: a = standardized path from problematic smartphone use (W14) to mediator (W15). b = standardized path from mediator (W15) to outcome (W16). Indirect effect = a × b—95% Monte Carlo confidence intervals (MCCIs) based on 20,000 simulated draws. * 95% MCCI excludes zero (unadjusted); ** 95% MCCI excludes zero AND survives Benjamini–Hochberg false discovery rate correction at *q* = 0.05 across all 16 paths.

## Data Availability

The Panel Study on Korean Children (PSKC) data can be found at https://panel.kicce.re.kr (accessed on 4 May 2026); access requires registration and compliance with the data provider’s terms of use.
